# Author Correction: Increasing extreme melt in northeast Greenland linked to foehn winds and atmospheric rivers

**DOI:** 10.1038/s41467-023-44592-2

**Published:** 2024-01-03

**Authors:** Kyle S. Mattingly, Jenny V. Turton, Jonathan D. Wille, Brice Noël, Xavier Fettweis, Åsa K. Rennermalm, Thomas L. Mote

**Affiliations:** 1 Space Science and Engineering Center, University of Wisconsin–Madison, Madison, WI USA; 2 Institute of Earth, Ocean, and Atmospheric Sciences, Rutgers, the State University of New Jersey, Piscataway, NJ USA; 3 Climate System Research Group, Institute of Geography, Friedrich-Alexander University, Erlangen, Germany; 4 Arctic Frontiers AS, Tromsø, Norway; 5 Institut des Géosciences de l’Environnement, CNRS/UGA/IRD/G-INP, Saint Martin d’Hères, France; 6 Institute for Atmospheric and Climate Science, ETH Zurich, Zurich, Switzerland; 7 Institute for Marine and Atmospheric Research Utrecht, Utrecht University, Utrecht, the Netherlands; 8 Department of Geography, University of Liège, Liège, Belgium; 9 Department of Geography, Rutgers, the State University of New Jersey, Piscataway, NJ USA; 10 Department of Geography, University of Georgia, Athens, GA USA

**Keywords:** Cryospheric science, Atmospheric dynamics, Climate and Earth system modelling

Correction to: *Nature Communications* 10.1038/s41467-023-37434-8, published online 29 March 2023

The original version of this Article contained an error in the “Introduction”, which incorrectly read ‘The Greenland Ice Sheet (GrIS) has lost ~3.9 billion tons of ice since 1992.’ The correct version states ‘~3,900 billion tons’ in place of ‘~3.9 billion tons’. This has been corrected in both the PDF and HTML versions of the Article.

