# Supplementary material for: Increasing extreme melt in northeast Greenland linked to foehn winds and atmospheric rivers
Source: Nat Commun. 2023 Mar 29;14:1743. doi: 10.1038/s41467-023-37434-8 (PMC10060376; doi:10.1038/s41467-023-37434-8)
Supplement: Supplementary file 1 — Supplementary Information [file 41467_2023_37434_MOESM1_ESM.pdf]

Supplementary Information for:

Increasing extreme melt in northeast Greenland linked to foehn winds and atmospheric rivers

Kyle S. Mattingly*[1,2], Jenny V. Turton[3,4], Jonathan D. Wille[5,6], Brice Noël[7,8], Xavier Fettweis[8], Åsa K. Rennermalm[9], Thomas L. Mote[10]

[1] Space Science and Engineering Center, University of Wisconsin – Madison, Madison, WI, USA
[2] Institute of Earth, Ocean, and Atmospheric Sciences, Rutgers, the State University of New Jersey, Piscataway, NJ, USA
[3] Climate System Research Group, Institute of Geography, Friedrich-Alexander University, Erlangen, Germany
[4] Arctic Frontiers AS, Tromsø, Norway
[5] Institut des Géosciences de l'Environnement, CNRS/UGA/IRD/G-INP, Saint Martin d'Hères, France
[6] Institute for Atmospheric and Climate Science, ETH Zurich, Zurich, Switzerland
[7] Institute for Marine and Atmospheric Research Utrecht, Utrecht University, Utrecht, Netherlands
[8] Department of Geography, University of Liège, Liège, Belgium
[9] Department of Geography, Rutgers, the State University of New Jersey, Piscataway, NJ, USA
[10] Department of Geography, University of Georgia, Athens, GA, USA

* Corresponding author email address: ksmattingly@wisc.edu

Supplementary Table S1. Summary validation statistics for the range of "melt day" thresholds applied to polar Regional Atmospheric Climate Model version 2.3p2 (RACMO2) and Modèle Atmosphérique Régional (MAR) data for comparison with NASA MEaSUREs satellite passive microwave melt data.

Melt threshold (mmWE / day)	Metric	RACMO2	MAR
0.1	accuracy	92.24%	93.26%
1	accuracy	93.31%	94.21%
2	accuracy	93.86%	94.50%
5	accuracy	94.18%	94.43%
8.25	accuracy	93.17%	93.71%
10	accuracy	92.50%	93.24%
0.1	true positive rate	82.58%	82.17%
1	true positive rate	75.84%	74.61%
2	true positive rate	71.02%	69.74%
5	true positive rate	56.36%	57.18%
8.25	true positive rate	38.49%	44.24%
10	true positive rate	30.14%	37.77%
0.1	false positive rate	6.66%	5.47%
1	false positive rate	4.70%	3.57%
2	false positive rate	3.54%	2.69%
5	false positive rate	1.52%	1.34%
8.25	false positive rate	0.62%	0.67%
10	false positive rate	0.41%	0.46%
0.1	true negative rate	93.34%	94.53%
1	true negative rate	95.30%	96.43%
2	true negative rate	96.46%	97.31%
5	true negative rate	98.48%	98.66%
8.25	true negative rate	99.38%	99.33%
10	true negative rate	99.59%	99.54%

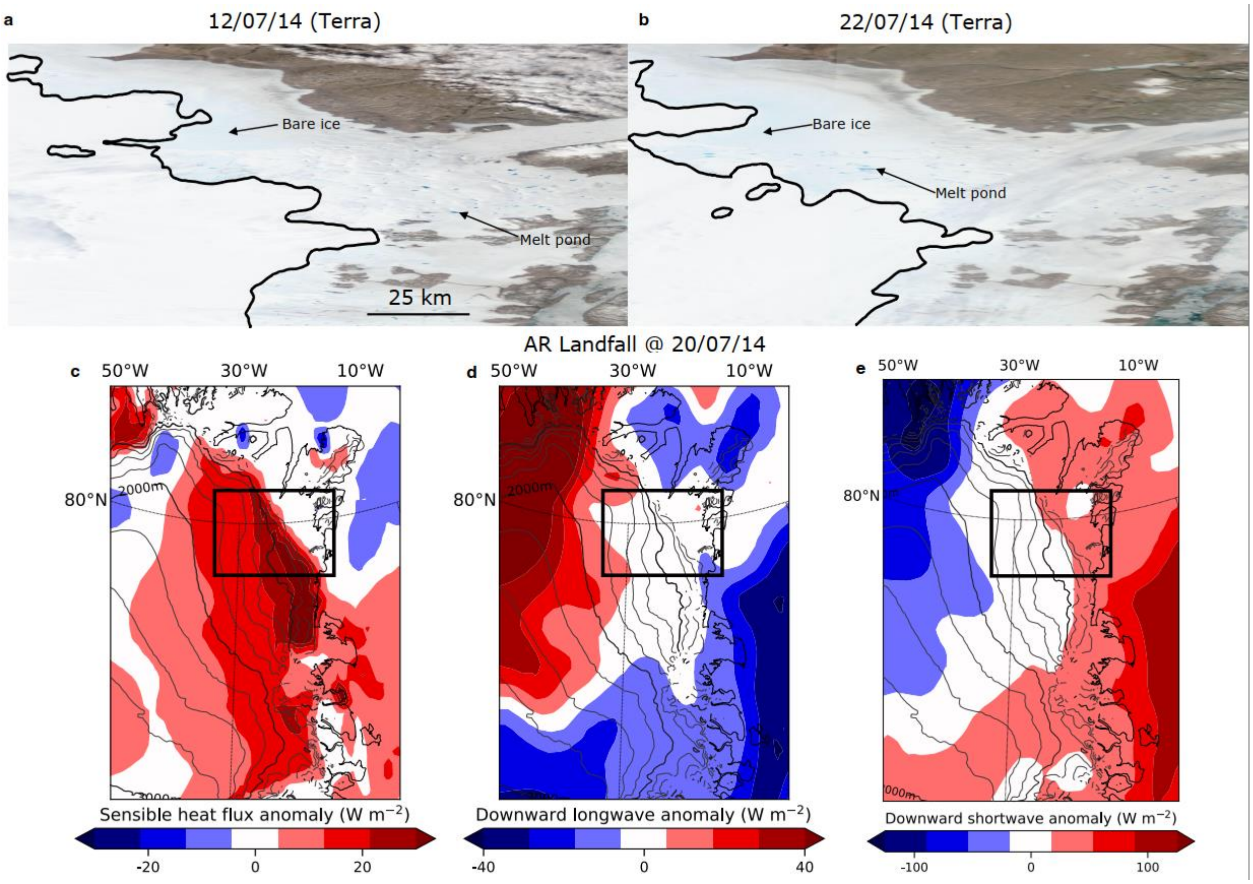

Supplementary Figure S1. Further details on the 20 July 2014 melt event. Moderate Resolution Imaging Spectroradiometer (MODIS) True Color satellite imagery from (a) 12 July 2014 and (b) 22 July 2014 showing the expanded bare ice and melt ponds following the passage of an atmospheric river (AR). Black line represents the approximate boundary between bare ice/melt ponds and untransformed snow. Anomalies of daily averaged (c) sensible heat flux (positive downward), (d) downward longwave radiation, and (e) downward shortwave radiation on the AR landfall date (20 July 2014) compared against the 1980–2020 average from ERA5 reanalysis. Boxes in (c–e) represent the approximate area of the satellite images in (a–b). Satellite images from the NASA MODIS instrument in (a) and (b) were obtained from the NASA Worldview application (https://worldview.earthdata.nasa.gov). We acknowledge the use of imagery from the NASA Worldview application (https://worldview.earthdata.nasa.gov), part of the NASA Earth Observing System Data and Information System (EOSDIS).

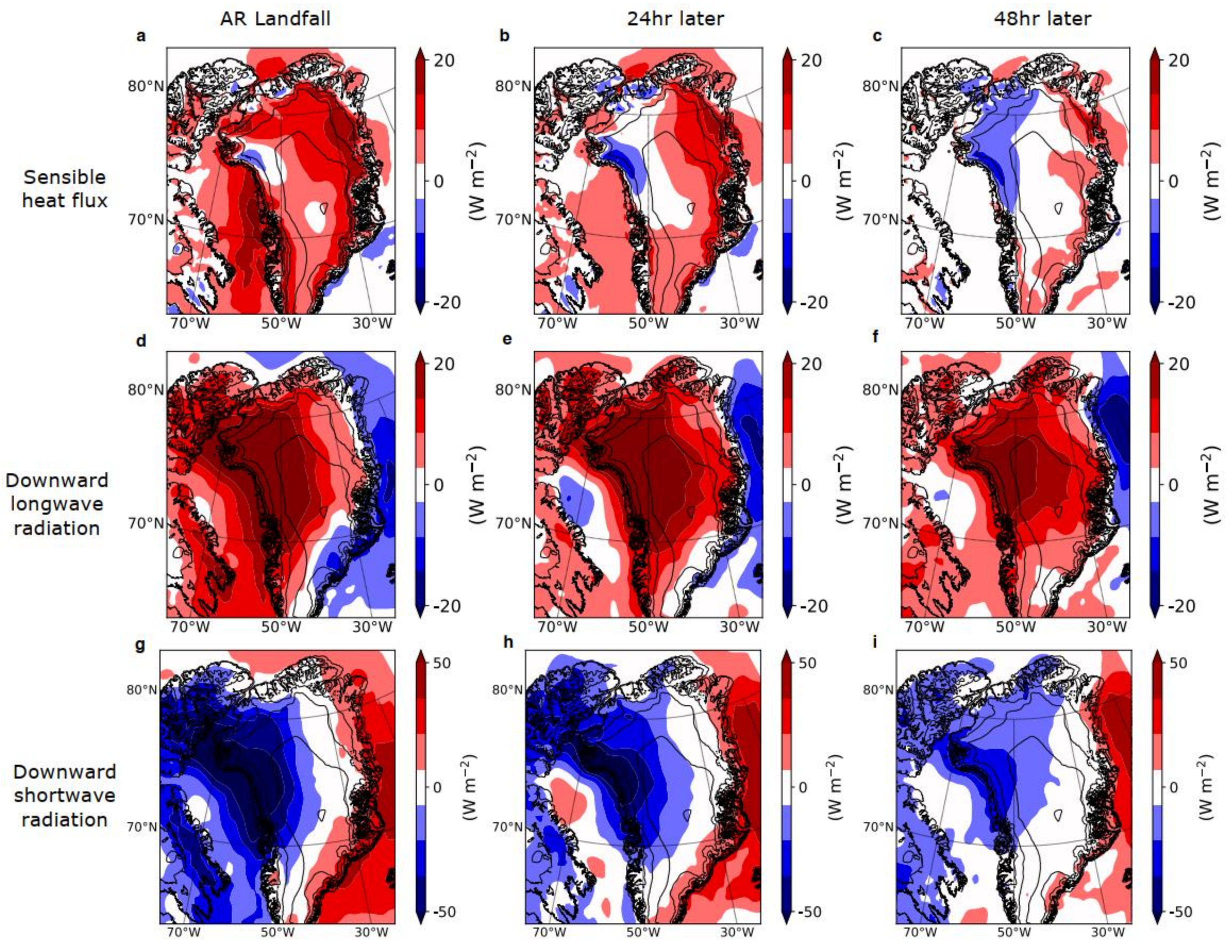

Supplementary Figure S2. Radiative flux anomalies during AR landfalls within the top 90th percentile of AR intensity according to the Mattingly et al., 2020 detection algorithm. (a–c) Composite sensible heat flux anomalies, (d–f) composite downward longwave radiation anomalies, and (g–i) composite downward shortwave radiation anomalies from the 1980–2020 ERA5 climatology that occurred (a), (d), (g) the same day, (b), (e), (h) 24 hours later, and (c), (f), (i) 48 hours later of an AR landfall in northwest (NW) Greenland (see the blue shaded region in Fig. 2g).

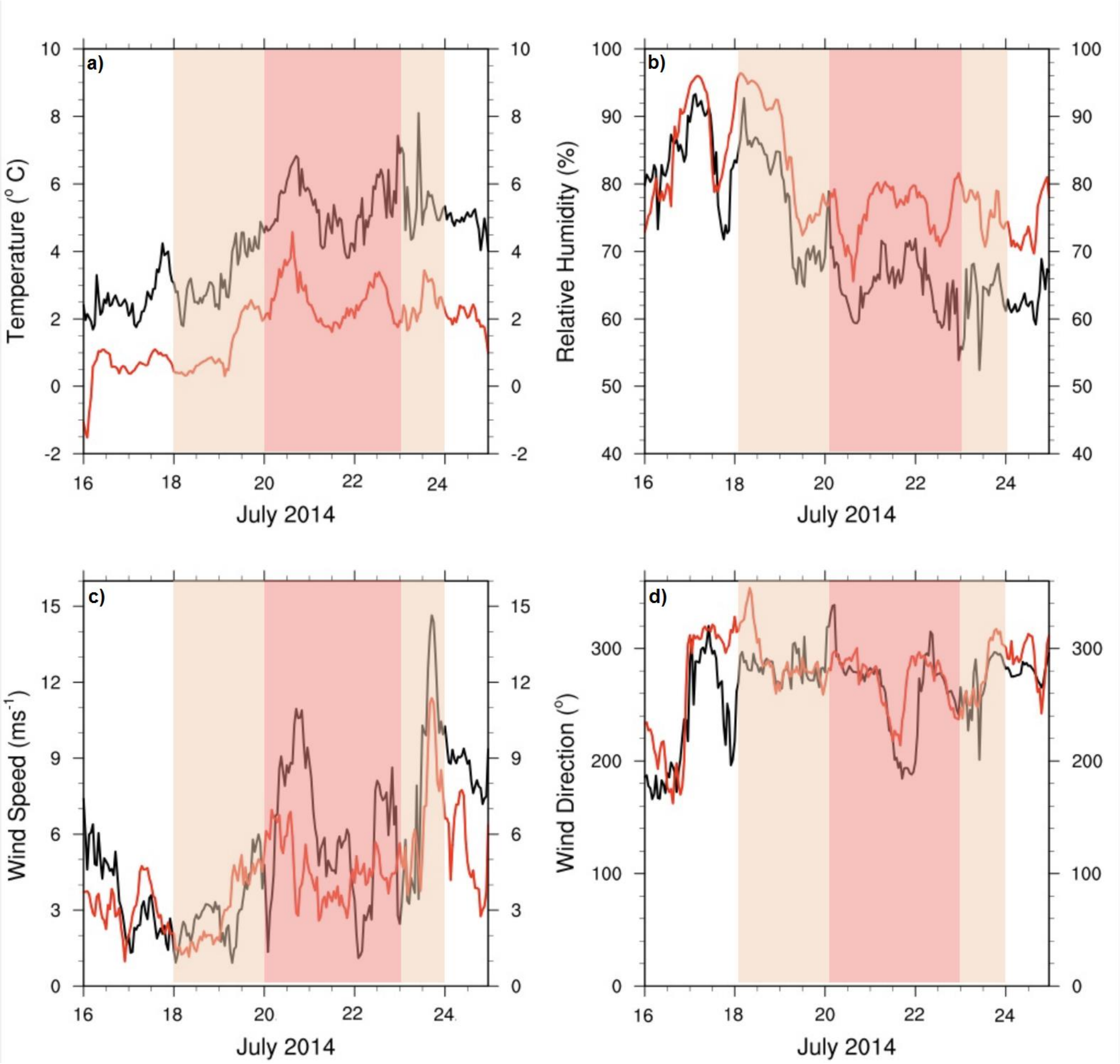

Supplementary Figure S3. Near-surface observations of temperature (a), relative humidity (b), wind speed (c) and wind direction (d) from the upper (red lines) and lower (back lines) Programme for Monitoring of the Greenland Ice Sheet (PROMICE) automatic weather stations (KPC_U and KPC_L respectively) from 16 July to 25 July 2014. Orange shading indicates the detection of an AR and red shading indicates the detection of a strong AR in NW Greenland using Modern-Era Retrospective analysis for Research and Applications, Version 2 (MERRA-2) reanalysis data.

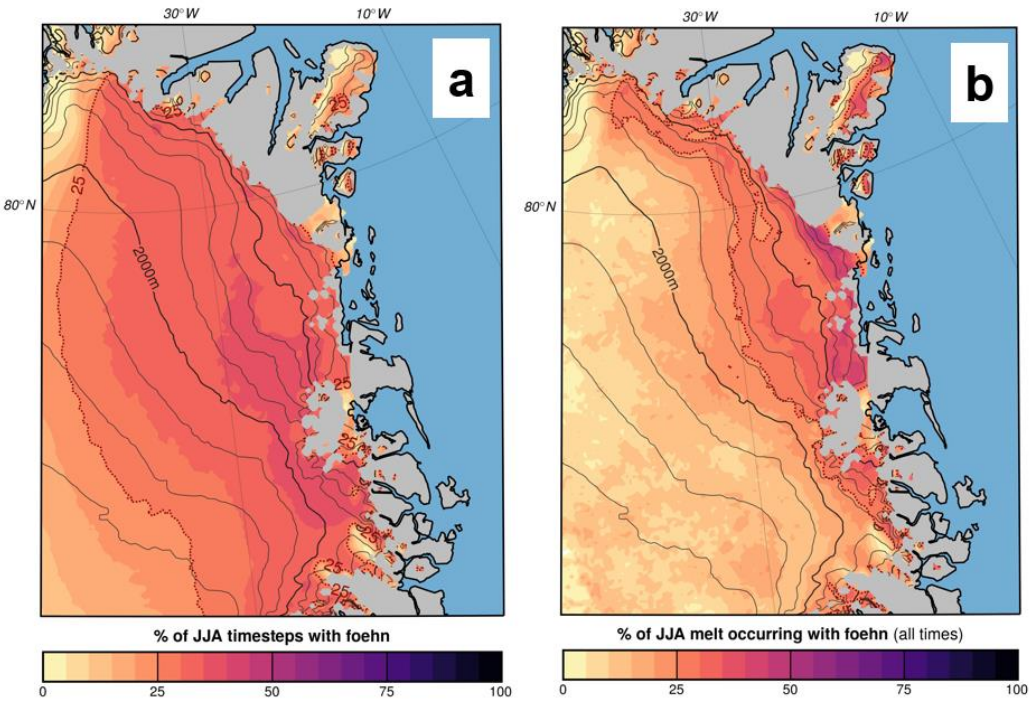

Supplementary Figure S4. (a) Polar Regional Atmospheric Climate Model version 2.3p2 (RACMO2) climatological percentage of summer (JJA) timesteps with foehn conditions. (b) RACMO2 climatological percentage of JJA melt occurring during foehn conditions, regardless of AR conditions.

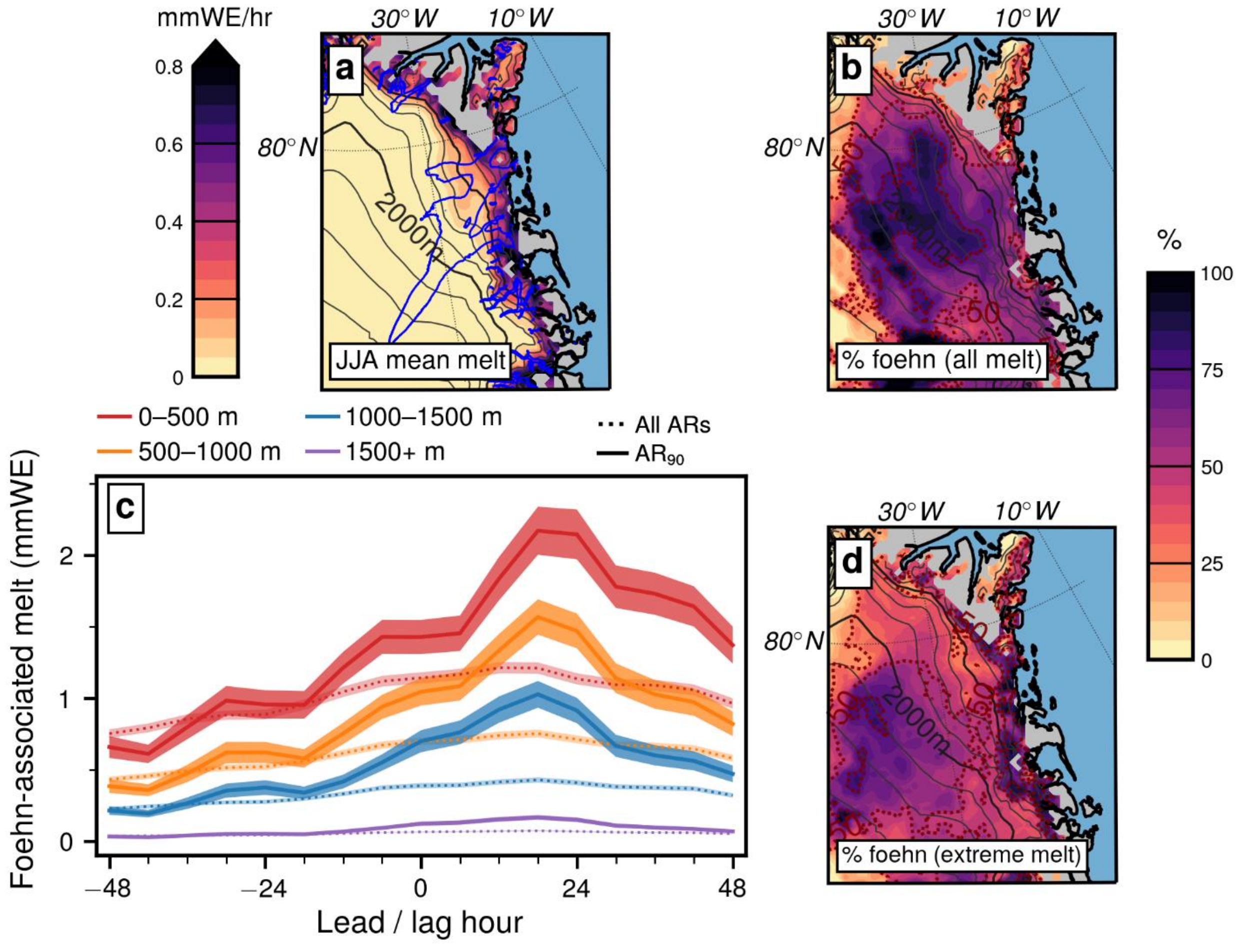

Supplementary Figure S5. As in Fig. 5 in the main text, but using MAR data. (a) Climatological mean hourly melt in northeast (NE) Greenland during JJA. (b) Percentage of melt coincident with foehn conditions during the 0–48 hour period after 90th percentile ARs (AR_{90}) in NW Greenland. (c) Temporal evolution of foehn-driven melt in NE Greenland in 500m elevation bands during the -48 to +48 hour period surrounding NW Greenland ARs. Lines are mean values and shading displays the standard error of the mean. (d) Map of percentage of extreme (> 99th percentile) melt coincident with foehn conditions.

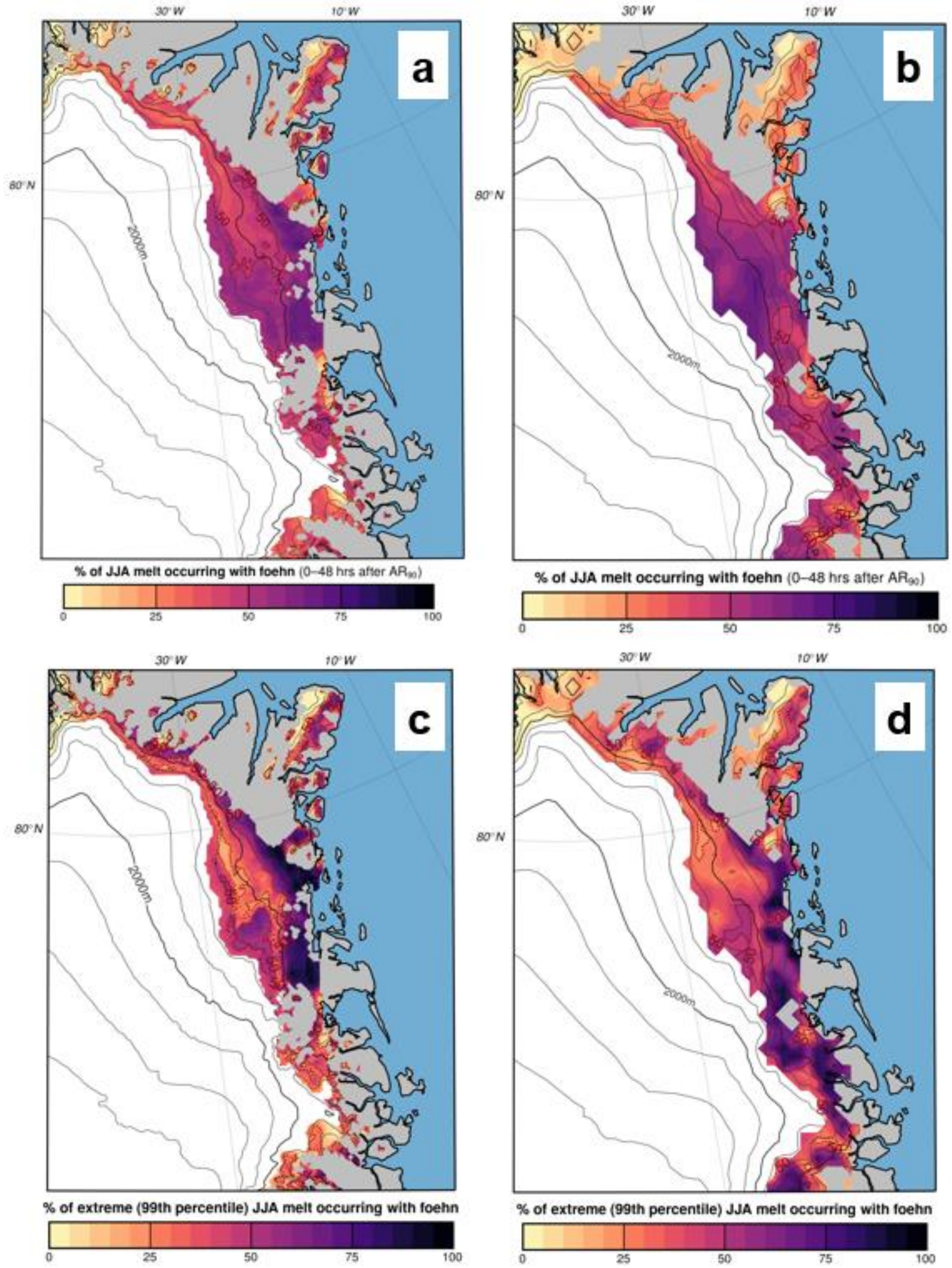

Supplementary Figure S6. As in (a) Fig. 5b (RACMO2), (b) Fig. S5b (MAR), (c) Fig. 5d (RACMO2), and (d) Fig. S5d (MAR), but with areas of JJA mean cumulative melt < 100 mmWE masked. Despite only covering 27.9% (32.9%) of the NE Greenland ice sheet domain in RACMO2 (MAR), these >= 100 mmWE melt areas account for 93.6% (96.4%) of total melt produced during an average summer.

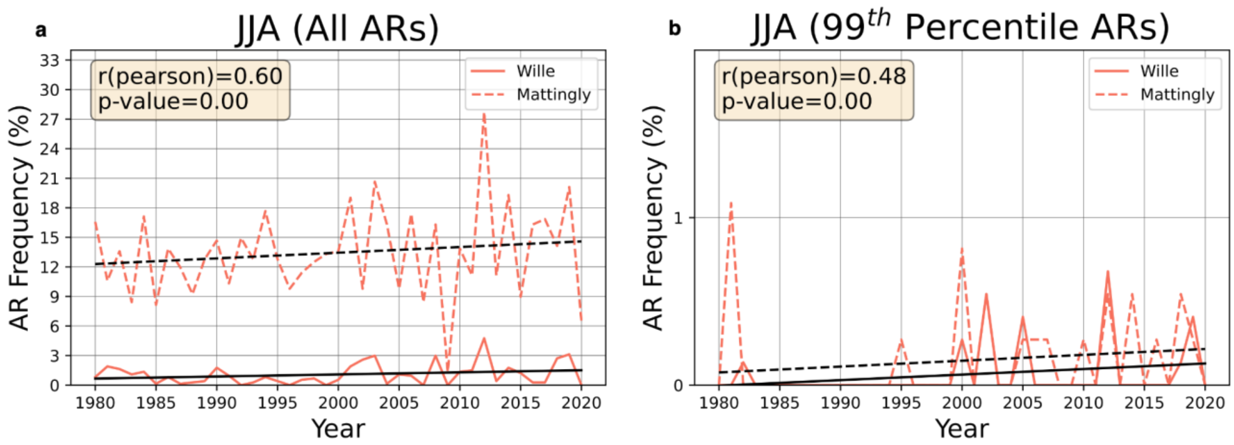

Supplementary Figure S7. Summer (JJA) AR frequency trends in NW Greenland (orange box in Fig. 1a) for (a) all ARs and (b) ARs in the 99th intensity percentile according to the AR detection algorithms in Wille et al., 2021 and Mattingly et al., 2020 applied to MERRA-2 data. AR intensity is derived from monthly climatological integrated water vapor transport (IVT) from 1980–2019.

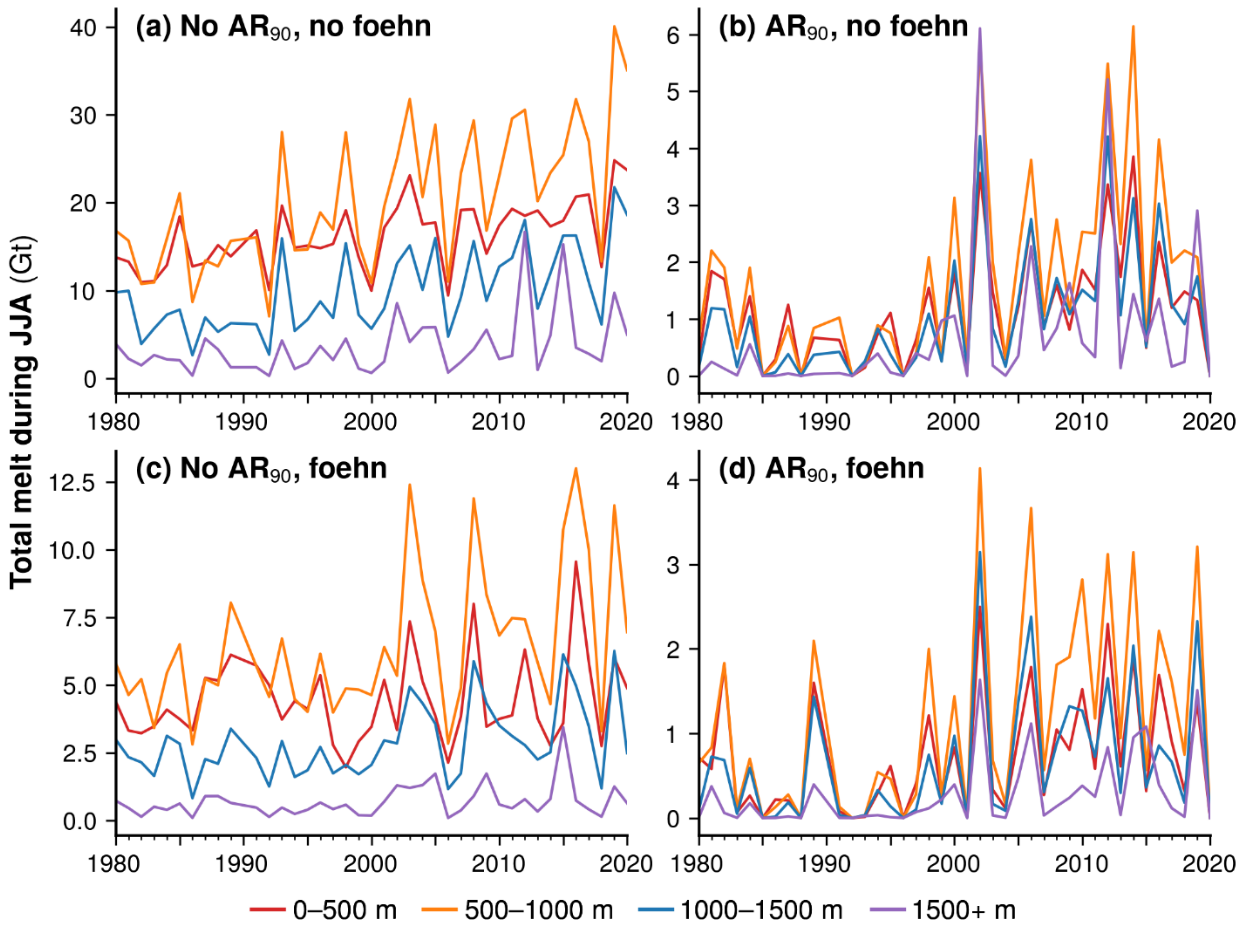

Supplementary Figure S8. Time series of total JJA melt (Gt) from RACMO2 integrated across all grid cells in NE Greenland elevation bands: 0–500m, 500–1000m, 1000–1500m, 1500+m. Melt sums are partitioned into the four possible combinations of AR_{90} and foehn conditions: (a) no AR with no foehn; (b) AR_{90} with no foehn; (c) no AR_{90} with foehn; (d) AR_{90} with foehn. Note that melt is attributed to AR_{90} conditions anytime a > 90th percentile AR was detected in the NW Greenland domain within the prior 48 hours.

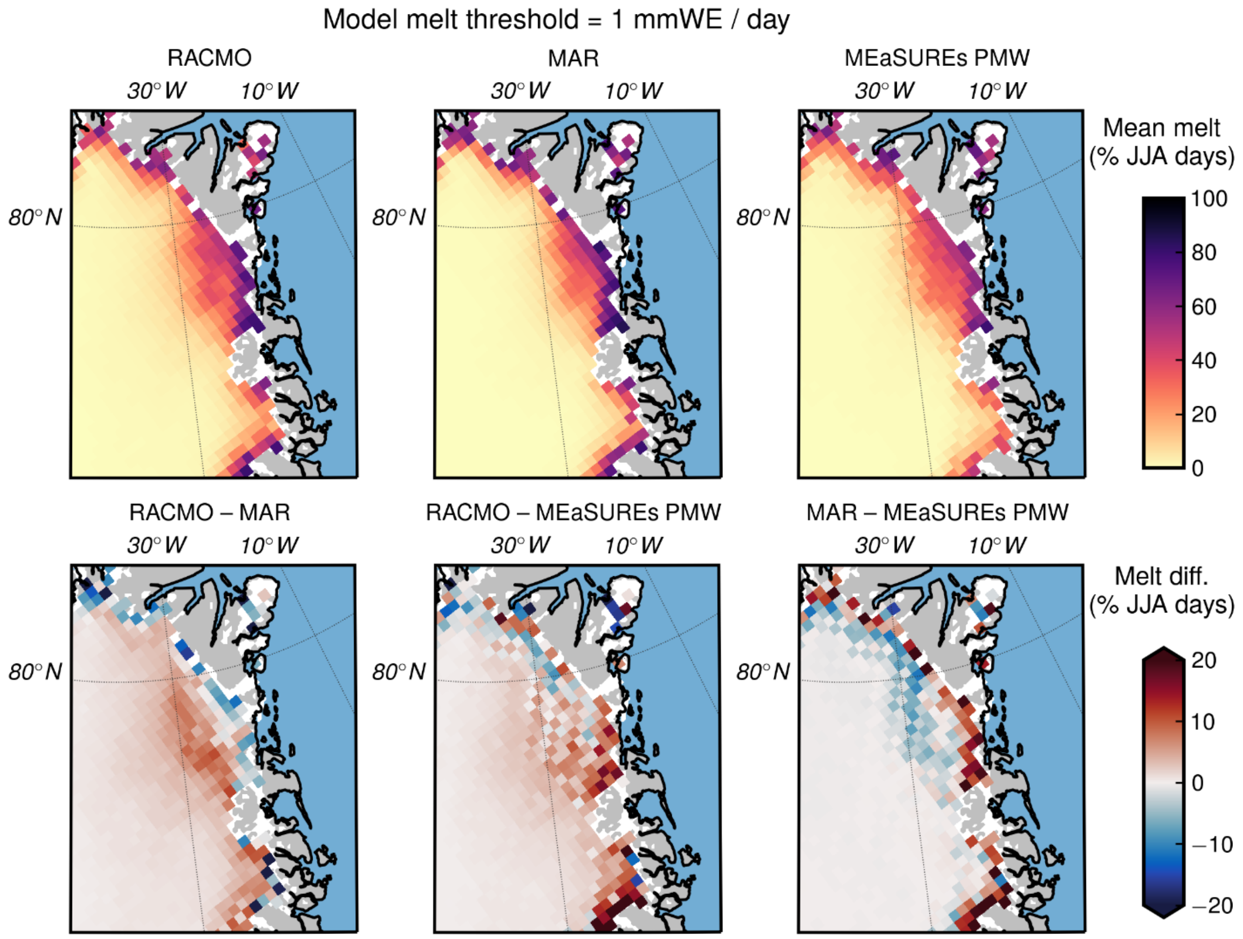

Supplementary Figure S9. Mean percentage of JJA days with melt from RACMO2 and MAR for a "melt day" threshold of 1 mmWE / day, with MEaSUREs passive microwave melt frequency included for comparison. Bottom row: differences in JJA melt day frequency between the datasets.

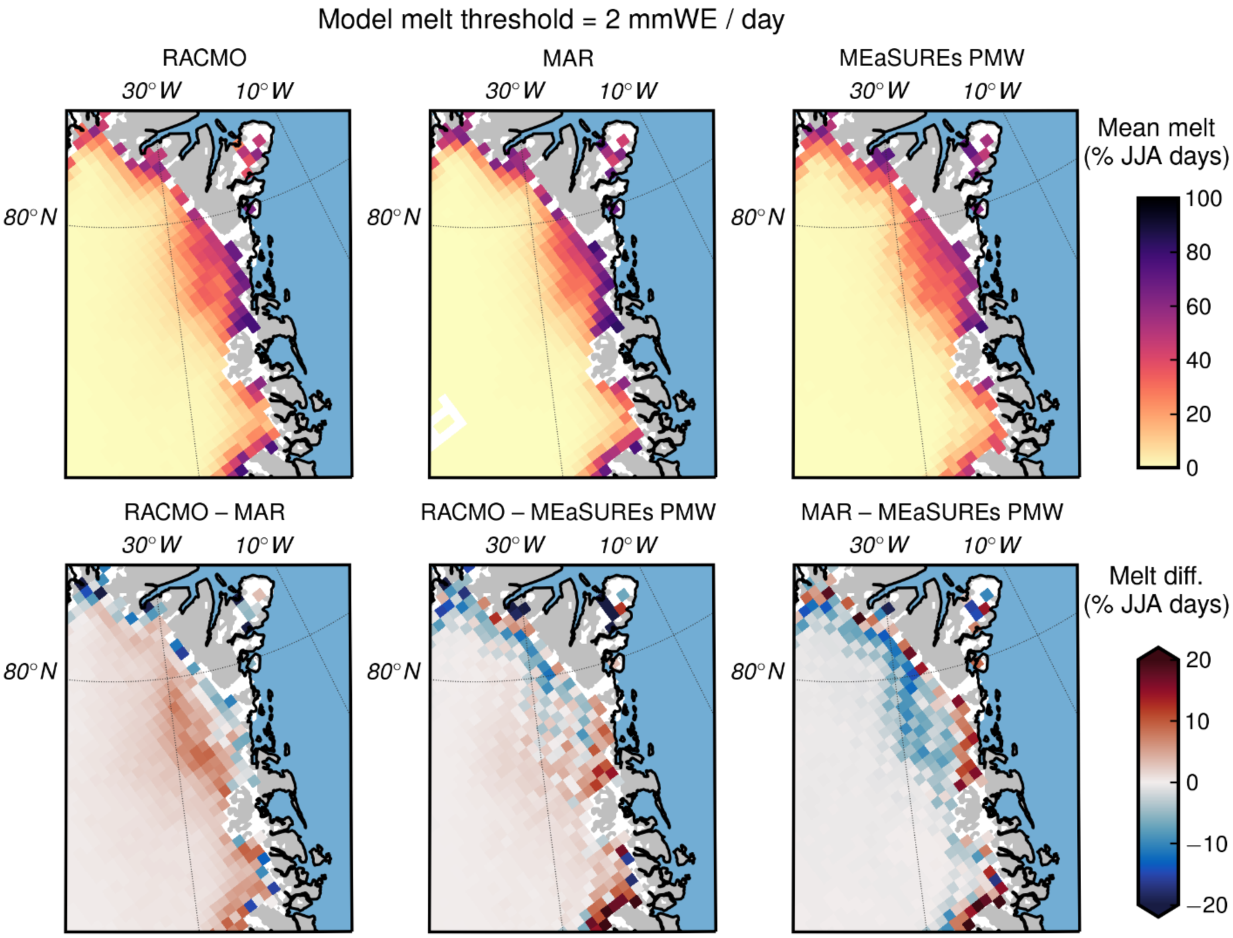

Supplementary Figure S10. As in Fig. S9 but for a "melt day" threshold of 2 mmWE / day.

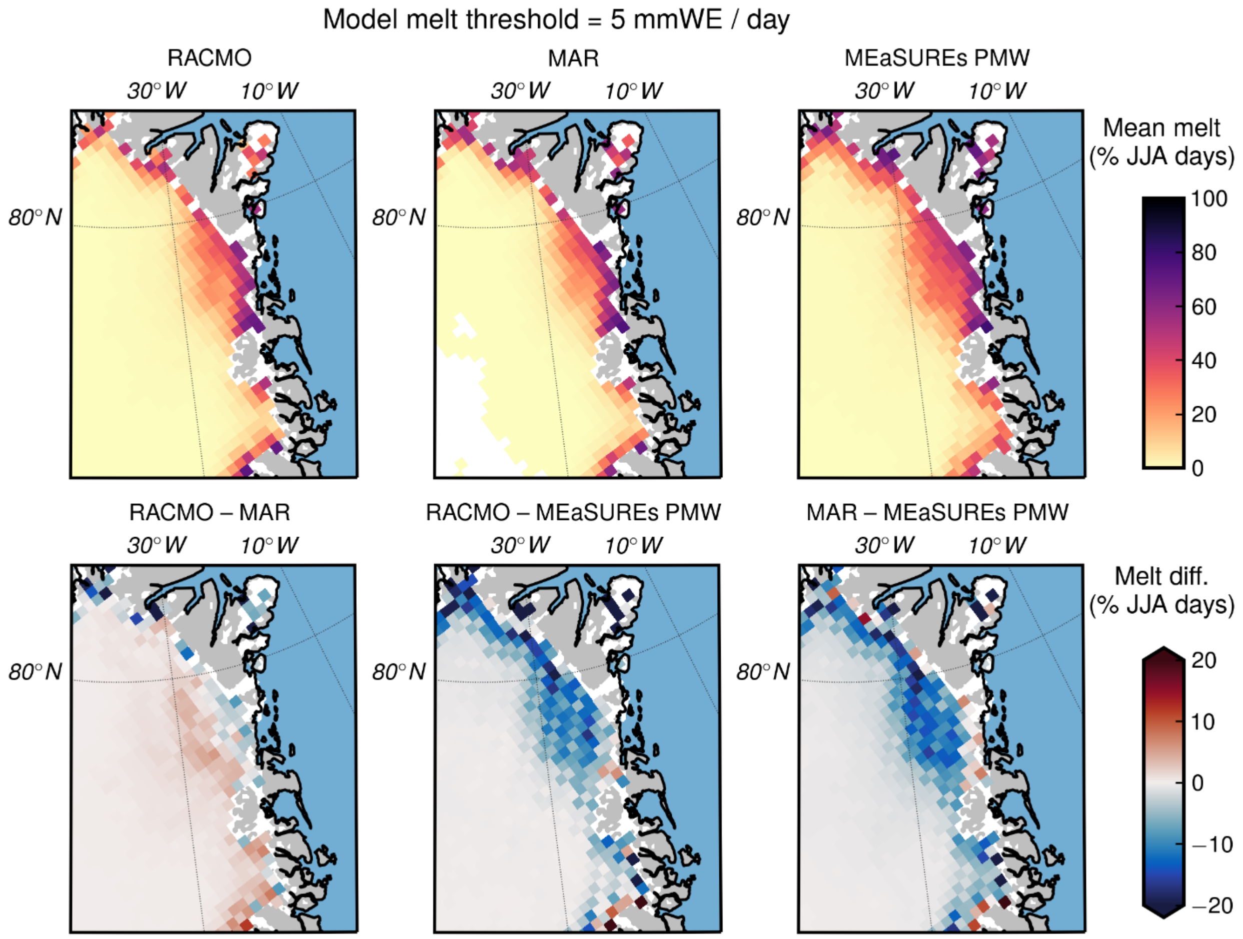

Supplementary Figure S11. As in Fig. S9 and Fig. S10 but for a "melt day" threshold of 5 mmWE / day.
